# Comparison of the Life History and Morphological Differences in Eight Korean Tiger Beetles Reared in the Laboratory to Develop an Ex Situ Conservation Method for the Endangered Tiger Beetle

**DOI:** 10.3390/ani15203032

**Published:** 2025-10-19

**Authors:** Deokjea Cha, Jong-Kook Jung, C. Barry Knisley

**Affiliations:** 1 Insect & Invertebrate Restoration Team, Division of Restoration Research, Research Center for Endangered Species, National Institute of Ecology, Yeongyang 36531, Republic of Korea; cj34gun@nie.re.kr; 2 Department of Forest Environment Protection, Kangwon National University, Chuncheon 24341, Republic of Korea; jkjung@kangwon.ac.kr; 3 Department of Biology, Randolph-Macon College, Ashland, VA 23005, USA

**Keywords:** tiger beetle, larval morphology, feeding behavior, life history

## Abstract

**Simple Summary:**

This study examined the ex situ conservation of eight Korean tiger beetle species through captive rearing. High mortality (37.5–80%) occurred during the transition from the pre-pupa to pupa stage, indicating that mass rearing is needed for effective population establishment. Reared-type adults were generally smaller than wild-type adults, and feeding habits, as well as the overwintering start point, varied by species. Overall, our results indicate that to develop a successful ex situ conservation method for endangered tiger beetles, it is crucial to adjust rearing methods to suit each species, rather than relying on general rearing methods. In addition, burrow entrance size was limited for species identification, while head and pronotum coloration were more useful.

**Abstract:**

Tiger beetles serve as bioindicators of ecosystem health but are under increasing threat from habitat loss and population decline. Ex situ conservation via captive breeding offers promise for species lacking viable wild populations. We evaluated laboratory rearing from egg to adult for eight Korean tiger beetle species to determine the developmental period per developmental stage, mortality rates, larval burrow entrance size, and head–pronotum morphological characteristics under controlled laboratory conditions. High mortality (37.5–80%) occurred during the transition from the pre-pupa to pupa stage, suggesting that mass larval production is needed to offset losses. Reared-type adults of most tiger beetle species tended to be smaller in body length than wild-type adults. Species-specific behaviors (e.g., feeding habits in *Cephalota chiloleuca*) and the overwintering times of spring–fall and summer species are different, indicating that uniform rearing protocols are suboptimal. Our findings suggest the importance of species-specific adjustment of rearing methods (feeding frequency, overwintering timing) to increase the success of ex situ conservation methods for tiger beetles. In addition, the larval burrow entrance size offered limited utility for species identification in mixed-species habitats, whereas the color of the head and pronotum was considered helpful in identifying some tiger beetle species.

## 1. Introduction

There are approximately 2600 species of tiger beetles known worldwide [1], and their habitats range from desert [2] and riverine [3] to salt marshes [4] and coastal areas [5], and they even include extremely hot spring regions [6]. Tiger beetles are considered a flagship species, providing indicators of their habitat environment due to their ecological characteristics, which include their habitat specificity [7]. Because adults and larvae reside in confined areas within their habitats, they are highly vulnerable to rapid environmental changes. Consequently, many tiger beetles are designated as endangered species and are protected [8]. In order to prevent tiger beetle population decline, conservation strategies primarily involve relocating and reinforcing larvae from healthy habitats to disturbed habitats, thereby strengthening the population [9]. Alternatively, captive propagation and larvae release strategies are implemented, involving capturing a small number of adults from healthy habitats and then mass-producing larvae, releasing them to a suitable habitat to establish a population [10,11].

However, when healthy populations are unavailable and sufficient larvae or adults of tiger beetles cannot be collected from the habitat for the aforementioned conservation strategies, in situ approaches become impractical. In such cases, ex situ conservation (i.e., captive breeding) based on the sanctuary concept can serve as an alternative approach to maintain and conserve target tiger beetle species in a laboratory environment. For this purpose, first, obtaining basic information on the ecological characteristics and life history of the target tiger beetle species is crucial [12]. Unfortunately, this approach is likely to be more successful in insect species with high egg production and survival rates, such as fruit fly and mealworm beetles. Maintaining a healthy population of insects through multiple generations with captive breeding is a difficult process, in part because long-term captive breeding may result in genetic deterioration [13], a decrease in fertility [14,15], and behavioral [16] and morphological [17] changes.

Until the early 2000s, a total of 18 species of tiger beetle were reported to inhabit South Korea [18]. However, recent exclusions due to misidentification (*Cicindela japana* Motschulsky, 1857 [19]; *Callytron nivicincta* Chevrolat, 1845 [20]; *Calltyron yuasai* subsp. *yuasai,* Nakane, 1955 [21]), regional extinction (*Cicindela coerulea* subsp. *nitida* Lichtenstein, 1796 [22]), and the addition of unrecorded species (*Cylindera kaleea* Bates, 1866 [23]), only 16 tiger beetle species are currently known to occur in South Korea. Among these are four summer species (i.e., tiger beetles that are active as adults in summer, lay eggs, and die that year) that are regionally red listed: *Cicindela* (*Abroscelis*) *anchoralis* Chevrolat, 1845 [20] (Critically Endangered); *Calomera brevipilosa* Horn, 1908 [24] (Vulnerable), *Cicindela obliquefasciata* Adams, 1817 [25] (Vulnerable); and *Chaetodera laetescripta* Motschulsky, 1860 [26] (Least Concern), due to habitat destruction and population declines [27]. While general habitat monitoring has been conducted for these endangered tiger beetles, research on basic ecological information, including life cycles, necessary for population restoration, has not been conducted. Thus, this preliminary study was conducted to assess the feasibility of rearing Korean tiger beetles through captive breeding under laboratory conditions and to identify key rearing processes essential for developing ex situ conservation methods for endangered tiger beetle species. In addition to studying ex situ conservation methods, we analyzed the size of larval burrow entrances and the head with the pronotum color of larvae to determine whether habitat monitoring is possible during the non-adult activity period.

## 2. Materials and Methods

**Figure 1:**
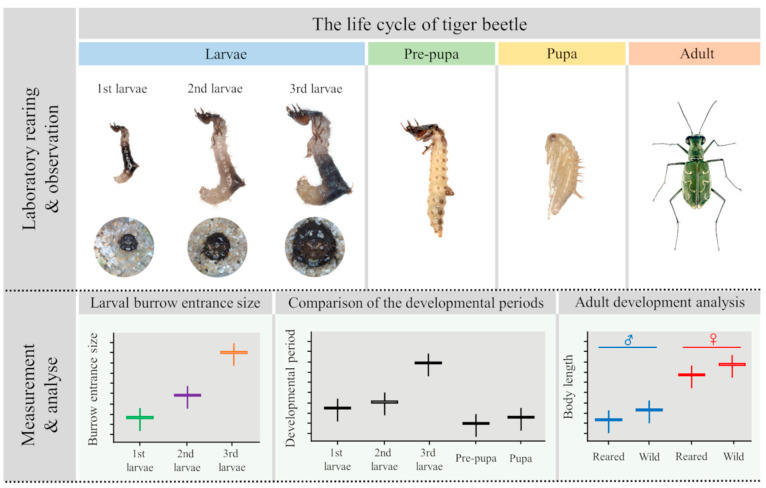
Diagram illustrating the main observations in this study of the life cycle of the tiger beetle, identifying developmental periods at different stages of development in propagation populations under laboratory conditions.

In this study, we reared four spring–fall species (*Cicindela transbaicalica* Motschulsky, 1844 [28]; *Cicindela gemmata* Faldermann, 1835 [29]; *Cicindela lewisii* Bates, 1873 [30]; *Cicindela chinensis* DeGeer, 1774 [31]) and four summer species (*Cicindela elisae* Motschulsky, 1859 [32]; *Cephalota chiloleuca* Fischer, 1820 [33]; *Myriochila specularis* Chaudoir, 1865 [34]; *Chaetodera laetescripta* Motschulsky, 1860 [26]) of Korean tiger beetles, developing them from egg to adult in the laboratory to determine the required developmental time and mortality rates for each developmental stage. Furthermore, we compared the body sizes of wild and propagated adults to assess whether propagated adults developed properly under laboratory conditions. We also compared the larval burrow entrance size of each tiger beetle species and the differences in the head and pronotum color of the larvae to determine whether habitat monitoring was possible with these ecological and morphological characteristics during the period, excluding the adult activity period in the habitat (Figure 1).

### 2.1. Field Collection and Adult Rearing

This study was conducted from 2023 to 2024, with each tiger beetle species captured during the adult activity season (May to June). To prevent over-collection by insect collectors, our capture sites for each tiger beetle species were approximately described rather than providing the exact habitat location. The number and habitat location of each species captured are described in Supplementary Material Table S1. Each tiger beetle was captured using an insect net at the field sites and transferred to an adult rearing jar (120 mm in diameter and 80 mm in height) filled with habitat soil pressed down to a height of 5 cm. The adult rearing method was modified from that used in a previous study [11]. All adult tiger beetles were individually reared in the adult rearing jar containing flattened surface habitat soil at a height of 5 cm. Each adult rearing jar was maintained in a controlled room, with a temperature of 24 to 25 °C, a relative humidity of 55 to 60%, and a 14:10 light–dark cycle. All adult tiger beetles were fed four workers of frozen Japanese wood ants (*Formica japonica*) and four frozen third-instar nymphs of two-spotted crickets (*Gryllus bimaculatus*) per day. The diet of adult tiger beetles was based on food sources identified in previous studies [35]. The day after feeding, any remaining diet debris was removed and replaced with fresh diet. Adult tiger beetles have behavioral characteristics of embedding their mouthparts in the soil to absorb moisture and laying eggs in soil with appropriate moisture conditions [36]. Thus, to maintain soil moisture suitable for oviposition within the adult rearing jar, water was sprayed onto the soil surface once daily.

To encourage copulation, a male was placed in the female rearing jar until copulation was observed. After copulation, the male was transferred back to the original rearing jar. This was repeated once a week until oviposition burrows were observed, after which the female was transferred to a new adult rearing jar. The rearing jars with oviposition burrows (and presumably eggs) were then stored under the same conditions as for adult rearing and were sprayed with water once a day to maintain soil moisture until the 1st-instar larvae hatched. All collected adults (wild type) were reared until they died and then transferred to 1.5 mL tubes and stored in a −20 °C freezer until measuring body length for comparison with the emerged adults (rear type).

### 2.2. Larvae Rearing

All larvae were reared under identical laboratory conditions as adults and individually because larvae exhibit cannibalistic behavior. The larval rearing tube was the bottom of a 50 mL conical tube (28 mm width × 120 mm height) with a cork lid (22 mm width × 25 mm height) inserted at the bottom of the tube, as described in a previous study [11]. The larval rearing tubes were filled with soil identical to the soil inhabited by the adults and compacted firmly. The soil surface was then leveled approximately 2 cm below the top of the tube and then compacted firmly with the fingers, and the soil surface was sprayed with water to prevent loosening of soil particles and to even out the surface.

When the 1st-instar larval burrows appeared in the adult rearing jar containing eggs, the soil was poured onto a stainless-steel tray, with the soil carefully separated to expose the first-instar larvae, which were then transferred to the soil surface of the larval rearing tubes. After transferring the larvae, a breathable 50 mL tube cap was placed on the rearing tube to prevent larvae from escaping. Once the 1st-instar larvae completed their burrowing, soil clumps from the larval burrowing were removed.

The larvae’s diet was modified from a previous study [35] and adjusted according to the developmental stage as follows: 1st-instar larvae were fed five frozen female fruit flies (*Drosophila melanogaster*) per day, 2nd-instar larvae were fed three frozen 3rd-instar cricket (*Gryllus bimaculatus*) nymphs per day, and 3rd-instar larvae were fed one frozen 4th-instar cricket nymph per day. After the prey items were thawed, they were attached to a feeding tool that consisted of a No. 0 insect pin taped to a bamboo chopstick, as described in a previous study [37]. The prey was pierced with the tip of the feeding tool and presented to the larva at the burrow mouth. The uneaten prey debris that larvae threw out of the burrow was removed once a day before feeding. When the soil in the larvae rearing tube was dry or severely contaminated by mold or green algae, the larvae were transferred to a new tube.

### 2.3. Overwintering

Excluding spring–fall species of tiger beetles (which developed into pre-pupae and pupae before winter), summer species of tiger beetles, whose 3rd-instar larvae had completed development, were placed in new larval rearing tubes. Once the larvae had completed their burrows, additional soil was added to fill the tubes, which were closed with breathable lids. The larval rearing tubes were inserted into tube racks and placed approximately 30 cm underground in the outdoor overwintering site. The tubes were then completely covered with river sand, ensuring no gaps were left between the tubes, and kept for an overwintering period that lasted 114 days, from November to March. The underground temperature in the outdoor overwintering site decreased from 15 °C to a low of 0 °C in winter and then rose to 15 °C in spring. After overwintering in March (1 March 2024 and 2025), the larval rearing tubes were removed from the overwintering site and transferred to laboratory conditions at 18 °C. The larvae were then transferred to new rearing tubes. The temperature in the laboratory was then increased by 2 °C every week until it reached 25 °C after 21 days, allowing for acclimatization after overwintering.

### 2.4. Pre-Pupa and Pupa Incubation

The initiation of the pre-pupal stage was indicated when the larvae constructed a pupal chamber and began peristalsis (dorsal–ventral movement). In order for pupation to be observed, the pre-pupal larvae were transferred to an artificial pupal chamber created by pressing an index finger 3 cm below the soil surface to create a 2.5 cm long × 2 cm wide chamber at a 30° angle. After creating the artificial pupal chamber, the soil surface was thoroughly watered to prevent it from collapsing due to drying. The pre-pupa was transferred using a reagent spoon and placed in the artificial pupal chamber with the head of the pre-pupa facing the soil surface and the abdomen oriented downward. The pre-pupae were then incubated under the same rearing conditions as adults, and water was sprayed on the walls of the rearing jar containing the artificial pupal chamber once a day to prevent drying. The pupae that had eclosed from the pre-pupae were kept under the same conditions and methods until they emerged as adults.

### 2.5. Observation Criteria for Developmental Stages

Hatching of 1st-instar larvae was impossible to observe due to the eggs being buried underground. Instead, the time from the formation of the 1st-instar larval burrow entrance was used as the start point for the 1st larval stage. Molting from 1st-instar larvae to 2nd-instar larvae occurs within burrows and cannot be observed, making it impossible to use the molting point as the endpoint. Instead, the time from the formation of the 2nd-instar larval burrow entrance was used as the endpoint for the 1st-instar larval stage. These same criteria were used to determine the times for the 2nd- and 3rd-instar larvae. However, the endpoint of the 3rd-instar larval stage was based on the transition point from 3rd-instar larva to pre-pupa. The developmental period of the 3rd-instar larvae was measured by dividing it into the developmental period for 3rd-instar larvae including the overwintering period, and the developmental period for 3rd-instar larvae excluding the overwintering period. The starting point of the pre-pupal stage was defined as the point when the 3rd-instar larvae, after creating a pupal chamber, were unable to use their legs and were moving in a peristaltic manner, and the abdominal area below the median hook was not folded. The endpoint of the pre-pupal stage was determined based on the time of molting into a pupa, while the pupal period is from the time the beetle molts from the pre-pupal stage to the time it emerges as an adult. Each developmental period was determined in days and recorded as mean and standard deviation values.

### 2.6. Measurement of Adult Body Length

Adult insect body size is affected by nutritional intake [38] and temperature conditions [39] during the larval stage. Thus, to determine whether the diet and quantity provided to larvae negatively affected the development of reared-type adults, the body sizes of wild-type and reared-type adults were measured and compared. After the adults died, they were frozen at −20 °C, and their body length was measured. To reduce individual body length measurement errors, all adults were measured with their heads, thoraxes, and elytra aligned. The mandibles (jaws) were excluded due to their significant differences in length between the closed and open positions. Body length was measured from the point of the frons (forehead) to the tip of the elytra using a vernier caliper (Absolute 500-181-30, Mitutoyo, Kawasaki-shi, Japan) capable of measuring to two decimal places.

### 2.7. Measuring the Diameter of the Larval Burrow Entrance and Photographing the Larval Head and Pronotum

Comparing the diameter of larval burrow entrances is the most useful method for determining larval developmental stages without removing them from their burrows [40]. In this study, we measured the diameter of larval burrow entrances from the three larval instars to determine whether burrow diameters can be used to differentiate species within the habitat. Diameters of the larval burrow entrance in larvae rearing tubes were measured under a digital-camera-equipped microscope (SMZ 745T with MSIP-REI-OB2-Truechrome IIs, Nikon, Tokyo, Japan) with a measurement program (TCapture, Tucsen, Fuzhou, China). In addition, we photographed the color and pattern of the head and pronotum of the larvae of the eight species under a microscope to determine if they were useful for species identification.

### 2.8. Data Analyses

We used the non-parametric Kruskal–Wallis test to compare the diameters of the larval burrow entrance because values were not normally distributed (Shapiro–Wilk tests, *p* = 0.05), and the variances were unequal (Bartlett tests, *p* = 1.5 × 10^−4^) following Dunn’s post hoc test, when statistical differences were observed between tiger beetle species. The body length values of wild-type adults and reared-type adults were compared using a two-sample *t*-test for each sex. The above two statistical analyses were conducted using R version 4.5.1 [41].

## 3. Results and Discussion

### 3.1. Comparison of Developmental Periods by Developmental Stage of Eight Species of Tiger Beetles

Our results found that among the spring–fall species, *C. transbaicalica* had the shortest larval development periods (from first to third instar), ranging from approximately 50 days in *C. transbaicalica* to approximately 90 days in *C. chinensis*. Excluding the overwintering period, summer species showed relatively short larval development periods in *C. elisae* (approx. 125 days) and *C. specularis* (approx. 127 days), while *C. chiloleuca* (approx. 187 days) and *C. laetescripta* (approx. 175 days) showed relatively long larval development periods. Although the developmental period of the third-instar larvae of *C. lewsii* was much shorter than that of the first- and second-instar larvae, most tiger beetle species required a longer developmental period as the larvae developed from the first- to third-instar larvae (Table 1).

The pre-pupal developmental period was the shortest for *C. chiloleuca*, averaging 9 days, while *C. gemmata* had the longest, averaging 22 days. In addition, the pupal development period was the longest for *C. lewsii* with an average of 21 days, and the shortest for *C. elisae* with an average of 15 days. The mortality rate was high in most of the tiger beetle species due to failure to molt from the pre-pupal to pupal stages (Table S2). Further studies are needed to elucidate whether the high mortality rate during this process is caused by transferring the pre-pupae to an artificial pupal chamber or whether it was caused by frequent molting failure during this developmental stage.

In a previous study, larval development of *Cicindela* (*Abroscelis*) *anchoralis* was delayed compared to other tiger beetle species due to its burrowing behavior, which involves creating multiple burrows, unlike other tiger beetle species [11]. In this study, it was confirmed that the larval development of *C. chiloleuca* was significantly delayed (average of 45 days for the first- and second-instar larvae) compared to other species because the first-instar larvae fed on prey items for no longer than one minute before throwing the partially eaten prey from the burrow. Most larvae of the other tiger beetles in this study stored prey at the bottom of their burrows after capture, fully consuming them, and then throwing the remaining debris out of the burrow (D.C.’s observation). This behavior of *C. chiloleuca* larvae resulted in less prey being consumed, leading to delayed larval development and higher mortality of first-instar larvae.

Previous laboratory propagation studies on *Cicindela chinensis japonica* showed that the developmental periods of the first-, second-, and third-instar larvae were, on average, 63 days, 28 days, and 1 year, respectively [35]. In that study, larvae appeared to have a delayed development time compared to *C. chinensis* in the present study. This difference appears to be the result of feeding once every two weeks and creating rearing conditions similar to natural environmental conditions (rearing larvae close to a window site) rather than feeding every day, with constant temperature, humidity, and light conditions in this study.

In addition, although it is not the result of artificial laboratory propagation, the developmental period of the larvae of the seven species of tiger beetles in their natural habitat was traced. Although there were differences depending on the species, the developmental periods of first-, second-, and third-instar larvae were found to be 1 to 2 months, 6 months, and 10 months on average, respectively [42]. In nature, the developmental period of the larvae of the tiger beetle is significantly longer than that of those reared in the laboratory. This is thought to be because the larvae do not actively hunt but rather settle in burrows, passively waiting for prey to pass by, preventing them from consistently hunting a constant amount of prey throughout their larval stage. This deprivation of prey in nature has been reported to delay larval development [35]. Moreover, a previous ex situ conservation study on another carnivorous beetle, *Dytiscus sharpi*, reported that the larval developmental period and the body size of reared adults varied depending on feeding conditions [43].

Therefore, to properly artificially propagate each tiger beetle in the laboratory, species-specific propagation methods would be applied by adjusting daily food quantity and the frequency of feeding according to the characteristics of each tiger beetle species after identifying the ecological and behavioral characteristics of the larval stage of the tiger beetle (e.g., daily intake; hunting and burrowing behavior).

### 3.2. Overwintering Requirements for Each Tiger Beetle Species

In South Korean tiger beetle species that begin the first instar in spring (April to May), the third instar larvae immediately create a pupal chamber after completing development and develop into a pre-pupal stage without overwintering. On the other hand, species that begin the first instar in the summer (June to August) do not create a pupal chamber after the third-instar larvae have completed development; rather, they block the burrow entrance and remain inside the burrow for overwintering. These behavioral patterns before the beetles enter overwintering correspond precisely to the two categories that divide the life history of the tiger beetle into the adult activity period (spring–fall species versus summer species) [44]. Therefore, it is necessary to adjust the target developmental stage of overwintering to reflect these different behavioral patterns.

In previous captive propagation studies of spring–fall species of tiger beetles, the reared adults were fed sufficiently before overwintering [45], but for summer species, the larvae are overwintered when they completed development and closed their burrows. This means that the timing of overwintering should be different for each tiger beetle species when artificially propagating. Therefore, to determine the exact time of overwintering, it is necessary to know if the target tiger beetle species is a spring–fall species or a summer species.

### 3.3. Comparison of Body Length of Reared-Type and Wild-Type Adult Tiger Beetles

**Figure 2:**
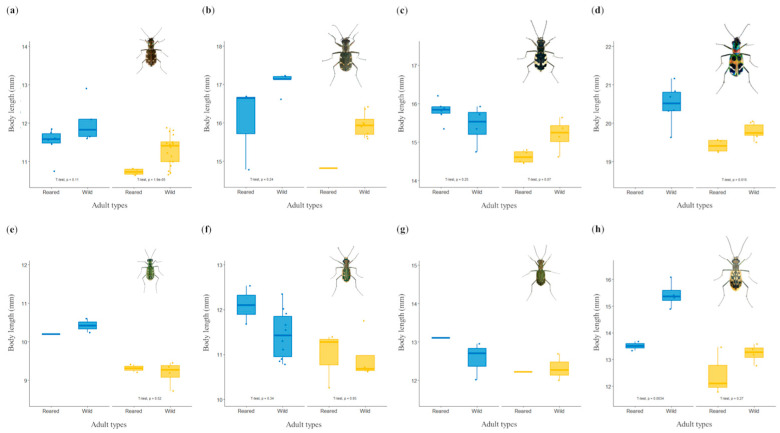
Comparison of body lengths of reared-type adults and wild-type adults from eight tiger beetle species to assess the artificial propagation under laboratory conditions. Each panel represents the species of the tiger beetle reared in this study. Panels from (**a**) to (**d**) represent the spring–autumn species (in order, *C. transbaicalica*, *C. gemmata*, *C. lewsii*, and *C. chinensis*), and panels from (**e**) to (**h**) represent the summer species (in order, *C. elisae*, *C. chiloleuca*, *M. specularis*, and *C. laetescripta*). The *x*-axis shows a comparison of the sexes (female: blue-colored, male: yellow-colored) of the reared-type adults and wild-type adults, and the *y*-axis shows the body length values of each adult. The analysis of body length differences by gender in each group used a two-sample *t*-test, and groups with insufficient data were excluded from the analysis.

While the body length of reared-type adults of *C. chiloleuca* (*t*-test, female, *p* = 0.34; male, *p* = 0.93), *C. lewisii* (*t*-test, female, *p* = 0.25; male, *p* = 0.07), *C. transbaicalica* (*t*-test, female, *p* = 0.11), *C. gemmata* (*t*-test, female, *p* = 0.24), *C. elisae* (*t*-test, male, *p* = 0.52), and *C. laetescripta* (*t*-test, male, *p* = 0.27) did not significantly differ from that of wild-type adults, some reared-type adults of *C. transbaicalica* (*t*-test, male, *p* = 1.9 × 10^−5^), *C. chinensis* (*t*-test, male, *p* = 0.015), and *C. laetescripta* (*t*-test, female, *p* = 3.4 × 10^−3^) tended to be smaller than their wild counterparts (Figure 2). Since the number of reared-type adults in some species was limited, statistical comparisons between reared-type and wild-type adults were not always possible (Tables S1 and S2). Future studies should determine whether the difference in body size between reared-type and wild-type adults results from sampling limitations or rearing methods, which will require analyses based on a larger number of individuals.

Previous studies have found that temperature [39,46] and food intake [38,47] were the factors that caused a decrease in adult size during laboratory rearing. To establish these species-specific rearing conditions, it is necessary to conduct a preliminary investigation of temperature variations (surface temperatures for adults and subsurface temperatures for larvae) within the habitat during larval and adult activity. It is also necessary to select an appropriate amount of prey for each larval stage. Finally, as described above, behavioral characteristics, such as the feeding behaviors (prey storage) that affect development, should be identified and adjusted for each species.

### 3.4. Evaluation of Larval Burrow Entrance and Head with Pronotum for Habitat Monitoring

**Figure 3:**
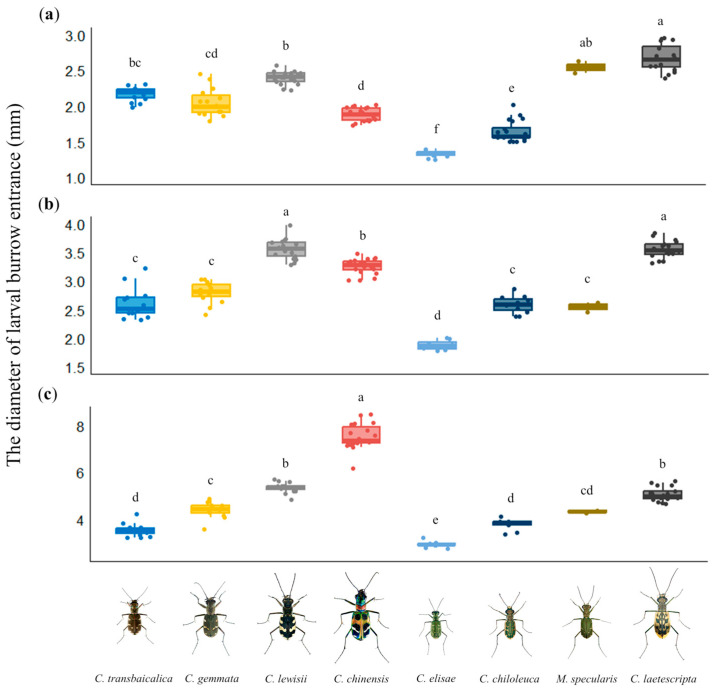
Comparison of burrow entrance diameter from larvae of eight tiger beetle species. The burrow entrance diameter of the larvae was compared with the mean value of the burrow entrance diameter of the 1st-instar (**a**), the 2nd-instar (**b**), and the 3rd-instar (**c**) larvae of each tiger beetle species. The different letters above bars indicate statistical difference at the 95% confidence level based on Dunn’s post hoc test.

**Figure 4:**
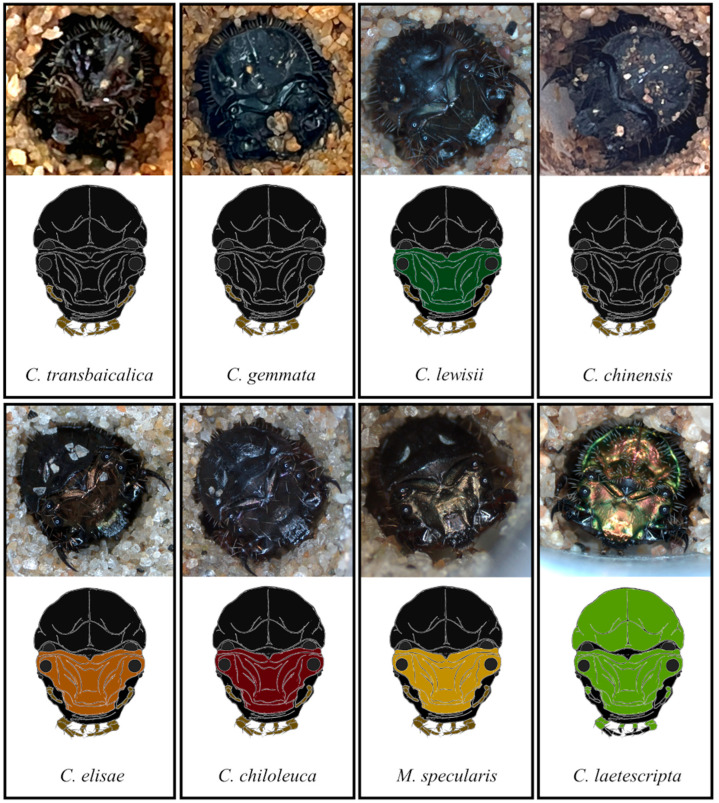
Comparison of the head and pronotum from larvae of eight tiger beetle species. The color and morphology of the head and pronotum from larvae remain almost unchanged as they develop from the 1st instar to the 3rd instar, increasing only in size. Thus, the head and pronotum from 3rd-instar larvae from each tiger beetle species are represented for comparison. The upper part of each panel displays the head and pronotum of a larva observed under a microscope, and the lower part of the panel features a diagram illustrating the head and pronotum to aid understanding.

As in a previous study of larval burrow diameters [11], we found larval burrow diameters can be used to determine the larval instar of a species, but not for distinguishing different species occurring in the same habitat (Figure 3). In contrast, the color of the head and pronotum of the larvae was useful for distinguishing certain species. The head and pronotum were both black in three species (*C. transbaicalica*, *C. gemmata*, and *C. chinensis*), so these could not be distinguished. The other five species had different and/or contrasting colors of the head and pronotum and could be effectively distinguished (Figure 4). It was judged that the characteristics of the larval head and pronotum can be used as a classification key for habitat monitoring through the observation of larvae during periods when adults are not active, particularly in some species.

## 4. Conclusions

The results of this study of artificial propagation of eight species of Korean tiger beetle found that high mortality rates (37.5~80%) during transition from the pre-pupal to pupal stage will require mass rearing of rare and endangered species to produce sufficient adults for the effective establishment of field populations. Our study also found it necessary to determine species-specific rearing methods, including the temperature, food type and amount, and behavioral characteristics that affect development. Understanding conditions for overwintering that differ between spring–fall and summer species is especially important. Although larval burrow size provided limited information for habitat monitoring, it can be used to infer larval developmental stages. Moreover, differences in larval head and pronotum morphology may serve as useful diagnostic features for habitat monitoring of certain tiger beetle species.

## Figures and Tables

**Table 1:** Developmental times and mortality rates for all stages of eight species of laboratory-reared tiger beetles.

Species	Adult Emergence Season	Habitats	Measurements	1st-Instar Larvae	2nd-Instar Larvae	3rd-Instar Larvae	Pre-Pupa	Pupa
*Cicindela transbaicalica*	Spring–Fall	Riverine, brackish water zone, seashore	Developmental period (day) *	11.38 ± 3.24 (16)	11.06 ± 1.95 (16)	13.70 ± 3.30 (10)	10.30 ± 1.49 (10)	15.30 ± 2.26 (10)
			Mortality (%) **	-	-	37.5	37.5	37.5
*Cicindela gemmata*	Spring–Fall	Riverine	Developmental period (day)	11.86 ± 2.60 (14) †	18.42 ± 3.18 (12)	37.83 ± 21.89 (6)	22.33 ± 9.67 (6)	19.00 ± 1.63 (4)
			Mortality (%)	-	14.3	57.1	57.1	71.4
*Cicindela lewisii*	Spring–Fall	Seashore	Developmental period (day)	24.29 ± 6.55 (17)	23.88 ± 5.04 (17)	13.20 ± 1.93 (10)	14.30 ± 4.30 (10)	20.90 ± 1.37 (10)
			Mortality (%)	-	-	41.2	41.2	41.2
*Cicindela chinensis*	Spring–Fall	Mountain path	Developmental period (day)	16.50 ± 2.44 (20)	31.25 ± 12.64 (20)	43.50 ± 17.02 (4)	15.00 ± 2.94 (4)	20.25 ± 1.50 (4)
			Mortality (%)	-	-	80	80	80
*Cicindela elisae*	Summer	Salt marsh, seashore	Developmental period (day)	23.38 ± 6.39 (8)	22.75 ± 3.49 (8)	187.67 ± 12.86 (3) [80.67 ± 12.86] ‡ (3)	11.00 ± 0.00 (3)	15.00 ± 1.00 (3)
			Mortality (%)	-	-	62.5	62.5	62.5
*Cephalota chiloleuca*	Summer	Salt marsh	Developmental period (day)	45.18 ± 12.28 (11)	47.00 ± 14.01 (9)	209.50 ± 25.11 (6) [95.50 ± 25.11] (6)	8.83 ± 0.98 (6)	18.60 ± 3.13 (5)
			Mortality (%)	-	18.2	45.5	45.5	54.5
*Myriochila specularis*	Summer	Riverine, brackish water zone, salt marsh	Developmental period (day)	20.00 ± 4.24 (2)	25.00 ± 2.83 (2)	196.50 ± 10.61 (2) [82.50 ± 10.61] (2)	8.50 ± 2.12 (2)	18.50 ± 0.71 (2)
			Mortality (%)	-	-	-	-	-
*Chaetodera laetescripta*	Summer	Riverine	Developmental period (day)	19.94 ± 2.80 (18)	24.89 ± 7.32 (18)	243.38 ± 33.76 (8) [129.38 ± 33.76] (8)	11.43 ± 1.40 (7)	15.80 ± 2.05 (5)
			Mortality (%)	-	-	55.5	61.1	72.2

*: Unit (average ± standard deviation). **: Mortality was recorded as cumulative mortality rates starting from the 1st-instar larvae. †: Parentheses indicate the number of individuals. ‡: In the case of square brackets, the developmental period excludes the overwintering period.

## Data Availability

The data analyzed during this study are available in the Supplementary Materials.

## Supplementary Materials

The following supporting information can be downloaded at https://www.mdpi.com/article/10.3390/ani15203032/s1: Table S1: Location information of beetle collection and the body length recorded from wild adult tiger beetles. Table S2: Life history records of eight tiger beetle species reared under laboratory conditions.
